# Characterization of Functional Human Skeletal Myotubes and Neuromuscular Junction Derived—From the Same Induced Pluripotent Stem Cell Source

**DOI:** 10.3390/bioengineering7040133

**Published:** 2020-10-22

**Authors:** Xiufang Guo, Agnes Badu-Mensah, Michael C. Thomas, Christopher W. McAleer, James J. Hickman

**Affiliations:** 1 NanoScience Technology Center, University of Central Florida, 12424 Research Parkway, Suite 400, Orlando, FL 32826, USA; Xiufang.Guo@ucf.edu (X.G.); ab16ab@Knights.ucf.edu (A.B.-M.); michael.c.thomas.1@knights.ucf.edu (M.C.T.); 2 Biomolecular Science Center, Burnett School of Biomedical Sciences, University of Central Florida, Orlando, FL 32826, USA; 3 Hesperos Inc., 12501 Research Pkwy, Suite 100, Orlando, FL 32826, USA; cmcaleer@hesperosinc.com

**Keywords:** skeletal muscle, iPSC, human, in vitro, characterization, fiber type, function, neuromuscular junction

## Abstract

In vitro generation of functional neuromuscular junctions (NMJs) utilizing the same induced pluripotent stem cell (iPSC) source for muscle and motoneurons would be of great value for disease modeling and tissue engineering. Although, differentiation and characterization of iPSC-derived motoneurons are well established, and iPSC-derived skeletal muscle (iPSC-SKM) has been reported, there is a general lack of systemic and functional characterization of the iPSC-SKM. This study performed a systematic characterization of iPSC-SKM differentiated using a serum-free, small molecule-directed protocol. Morphologically, the iPSC-SKM demonstrated the expression and appropriate distribution of acetylcholine, ryanodine and dihydropyridine receptors. Fiber type analysis revealed a mixture of human fast (Type IIX, IIA) and slow (Type I) muscle types and the absence of animal Type IIB fibers. Functionally, the iPSC-SKMs contracted synchronously upon electrical stimulation, with the contraction force comparable to myofibers derived from primary myoblasts. Most importantly, when co-cultured with human iPSC-derived motoneurons from the same iPSC source, the myofibers contracted in response to motoneuron stimulation indicating the formation of functional NMJs. By demonstrating comparable structural and functional capacity to primary myoblast-derived myofibers, this defined, iPSC-SKM system, as well as the personal NMJ system, has applications for patient-specific drug testing and investigation of muscle physiology and disease.

## 1. Introduction

The skeletal muscle is the largest organ of the human body. Aside being responsible for all voluntary movement, skeletal muscle is an important regulator of whole body metabolism [1]. Developing in vitro systems to differentiate human skeletal muscle from stem cells is needed for tissue engineering, diagnostics and human-on-a-chip applications [2,3,4]. Pathogenic alterations in skeletal muscle physiology are also linked to poor prognosis in many human diseases, such as Duchenne muscular dystrophy (DMD) and Amyotrophic lateral sclerosis (ALS). However, the exact mechanism by which altered skeletal muscle function contributes to these diseases is undetermined. Thus, a reliable source of human subject-relevant muscle derived from stem cells is desired for tissue engineering, disease modeling and cell therapy.

Human pluripotent stem cells (hPSCs), including embryonic stem cell (ESC) and induced PSCs (iPSCs), possess unlimited proliferative potential while maintaining the ability to differentiate into any cell type, therefore representing an easy access, inexhaustible cell source. Human iPSCs can be generated from a variety of somatic cell types from healthy donors and patients, thus eliminating the ethical and technical hurdles when being used as a cell source. Compared to primary satellite cells, utilizing iPSCs as the source for muscle differentiation offers several advantages. Primary satellite cells are usually low in number in muscle, difficult to isolate, have limited proliferative capacity and lose in vivo regenerative potential upon ex vivo expansion [5]. Also, iPSCs are more amenable to genetic editing and can be derived from patient somatic cells, therefore holding great promise for cell replacement therapy. In addition, since multiple cell types can be generated from the same iPSC line, it is becoming more practical to develop patient-specific models not only for muscle, but also for muscle-related multi-organ systems, such as the neuromuscular junction. 

Multiple studies have reported successful skeletal myogenesis by utilizing hiPSCs as the source. Generally, three approaches have been investigated to date. The first is by introducing exogenous DNA into the iPSCs to force expression of transcription factors such as myoblast determination 1 (MyoD1), Paired box 3 (Pax3) and Paired Box 7 (Pax7) [6,7]. This approach demonstrated high efficiency in generating functional muscle cells, which is important for developing large scale drug screening platforms. However, it is genetically invasive and the induction process is relatively artificial, therefore it poses some concerns for the utilization of the generated cells, especially for in vivo cell therapy. The second approach tested was through the formation of embryoid bodies, in which other cell types can also be induced in addition to muscle cells [8]. This strategy makes use of natural development processes. However, it has low reproducibility since it exhibits poor control of the differentiation process. In addition, myogenic precursors are only one of the cell types induced and it usually relies on cell sorting to obtain an enriched population for muscle differentiation. A third approach guides the differentiation process by applying a series of factors to a monolayer of iPSCs [9]. This strategy recapitulates the in vivo developmental pathway of the muscle lineage, and allows fine control of the differentiation process, thus having the potential for better reproducibility and was the method utilized in this study. 

Preliminary characterization of the differentiated myofibers has been done in all the studies that reported successful myogenesis from human iPSCs. Demonstration of the expression of muscle filament proteins such as myosin heavy chain, actin or titin, in generated myotubes have been utilized as basic biomarkers for successful demonstration of the derivation of myofibers [8,10,11]. A study displayed Z-line–like structures in generated myofibers with EM [6]. Another study demonstrated the membrane currents induced by Ach in skeletal muscle cells with patch clamp electrophysiology [12]. Myofiber contractions under electrical stimulation and Ach-induced action potentials in iPSC-derived human skeletal muscle (iPSC-hSKM) have only been displayed in one study [13]. However, the collective differentiation protocols involved a number of undesired features such as mouse embryonic fibroblasts (MEF) feeder layers, EB formation, serum and conditioned medium from primary myoblast cultures, and co-culture with iPSC-derived human motoneurons (iPSC-hMNs). In general, characterization of iPSC-SKM is limited, either concerning the resultant skeletal muscle structure or its function. Recently, the generation of functional skeletal muscle tissue (3D) from human iPSC-derived has been reported [14]. Myogenic progenitor cells were induced via transient overexpression of Pax7 in paraxial mesoderm cells differentiated from hPSCs. The generated skeletal muscle tissues exhibited a positive force-frequency relationship and robust calcium transients in response to electrical or acetylcholine stimulation. However, the literature describing the properties of hiPSC derived-myofibers in 2D culture is still very limited and this knowledge gap hinders the application of iPSC-hSKMs in disease modeling. Especially, there is an increasing demand for patient-specific neuromuscular junction (NMJ) models, but with limited progress to date. The first published study that successfully established an NMJ co-culture by combining MNs and myotubes from the same donor human iPSC line demonstrated putative NMJs by morphological analysis in the absence of any functional evidence [13]. Functional NMJs derived from a single iPSC line has been reported consisting of MNs and myotubes in a differentiated complex tissue derived by temporarily expressing MYOD1 [15], and in a MN-SKM mixed co-culture of independently differentiated MNs and SKMs, where functional NMJ formation was evidenced for both by optogenetics [16].

This study, for the first time, demonstrated a functional NMJ platform in which MNs and SKM were independently differentiated from the same iPSC line, co-cultured in chemically and electrically isolated chambers, and functionally tested with electrical stimulation. The muscle cells were differentiated from human iPSCs by adapting the protocol reported by Chal [11], which mimics the natural differentiation pathway in an attached monolayer of cells in a feeder-free, serum-free system. The structure and function of the differentiated myotubes were characterized and compared to the myofibers derived from primary satellites. The structural and functional characterizations of these myofibers that were derived epigenetically from hiPSCs enabled the integration with motoneurons derived from the same line to establish a neuromuscular junction system. The integration of the BioMEMs chamber system with the iPSC technology enables a patient-specific NMJ model for high-content functional interrogation, tunable to chemical and genetic manipulations [17,18] as well as addition of other cell types. Therefore, the donor-specific NMJ system demonstrated in this study could provide a valuable platform for investigating the pathology of NMJ-related diseases and testing patient-specific therapeutics.

## 2. Materials and Methods 

### 2.1. Culturing and Expansion of hiPSC 

The human iPSC line (ND41865) was purchased from the Coriell Institute Stem Cell Biobank at NIH. The cells were expanded on a surface coated with Matrigel (Corning) in TesR ^TM^-E8 ^TM^ medium (Stemcell Technologies, Vancouver, Canada) by following the instructions described by the cell biobank. The iPSCs were expanded for up to 10 passages and cryopreserved in 10% dimethyl sulfoxide (DMSO) containing TesR ^TM^-E8 ^TM^ medium in liquid N_2_. Cells between passages 6 and 10 were utilized for differentiation. 

### 2.2. Differentiation of Myogenic Progenitorss and Functional Myofibers from hiPSCs

Myogenic progenitors were differentiated from hiPSCs according to the protocols described in [9,11]. Briefly, myogenic progenitors were generated through a multi-step small molecule differentiation protocol, and later subcultured in hSKM Growth Medium (Lonza, Basel, Switzerland). Myogenic progenitors were expanded, passaged and cryopreserved for subsequent use. For myofiber differentiation, passage 3 frozen myogenic progenitors were thawed in Lonza hSKM Growth Medium and plated onto glass coverslips coated with either 0.083 mg/mL Matrigel or 60 µg/mL Collagen I solution. Growth medium was refreshed every 2 days until confluency. Cultures were then switched to HI medium for 48 h, and then into N2 medium until terminal differentiation. In N2 medium, cultures were fed every 2 days by changing half of the medium. The age of the culture was counted from the day switching to HI medium. The components of HI and N2 medium were as described in [11]. HI medium is composed of DMEM/F12 (ThermoFisher Scientific, 21041-025, Waltham, MA, USA), 15% vol/vol Knockout Serum Replacement (ThermoFisher Scientific 10828-028, Waltham, MA, USA), 10 ng/mL hepatocyte growth factor (HGF) (Peprotech 315-23, Rocky Hill, NJ, USA), 2 ng/mL insulin-like growth factor (IGF) (Sigma I1271, St. Louis, MO, USA, 0.1 mM), β-mercaptoethanol (ThermoFisher Scientific 31350010), 1% MEM Non-Essential Amino Acids Solution (ThermoFisher Scientific 11140050) and 100 nM dexamethasone (Sigma D4902). N2 medium is composed of DMEM/F12 (ThermoFisher Scientific, 21041-025), 1% Insulin Transferrin Selenium (ITS) (ThermoFisher Scientific, 41400045), 1% N2 supplement (ThermoFisher Scientific, 17502048), 1% L-glutamine 20 mM (ThermoFisher Scientific, A2916801), and 100 nM dexamethasone (Sigma, D4902).

### 2.3. Immunocytochemistry

Cultures on coverslips were fixed in freshly prepared 4% paraformaldehyde in Phosphate Buffered Saline (PBS) (pH 7.2, without Mg^2+^, Ca^2+^) for 15 min. Cells were washed twice with PBS for 10 min at room temperature and then permeabilized with 0.1% Triton X-100/PBS for 15 min. Non-specific binding sites were blocked using Blocking Buffer (5% donkey serum plus 0.5% Bovine Serum Albumin (BSA) in PBS) for 1 h at room temperature. Cells were incubated with primary antibodies overnight at 4 °C. Following primary antibody incubation, cultures were washed with PBS 3X at 5, 10 and 15 min intervals with PBS. Cultures were incubated with secondary antibodies for 2 h at room temperature and then washed 3X with PBS for thirty minutes total. After staining, the coverslips were mounted using ProLong™ Gold Antifade Mountant with 4′-6-Diamidino-2-Phenylindole (DAPI) (Thermofisher Scientific, P36931). Primary antibodies used in this study were: mouse-anti-MyoD (abcam, ab16148, Cambridge, UK), rabbit-anti-Pax7 (abcam, ab34360), mouse-anti-Myosin Heavy Chain (MHC, F1.625, 1:10), mouse-anti-DHPRα1 (Dihydropyridine receptor α1, Millipore, Billerica, MA, USA, 1:500), and rabbit-anti-Ryanodine Receptor (Millipore, Burlington, MA, USA, 1:500). Phalloidin-568 (Invitrogen, Carlsbad, CA, USA, 1:200) was added to the secondary antibody solutions in order to facilitate actin filament visualization. The monoclonal antibody against Myosin Heavy Chain (MHC, F1.625, 1:10) was obtained from the Developmental Studies Hybridoma Bank which is under the auspices of the National Institute of Child Health and Human Development (NICHD) and maintained by the University of Iowa. Secondary antibodies used in this study were: donkey-anti-mouse-488 (Invitrogen, A-21202 1:250) and donkey-anti-rabbit-594 (Invitrogen, 1:250). All antibodies were diluted in Blocking Buffer. 

### 2.4. Electrophysiology

Electrophysiological properties of the human myotubes were investigated after ~10 days in N2 medium, using whole-cell patch clamp recording techniques [19]. The recordings were performed in a chamber located on the stage of a Zeiss Axioscope 2FS Plus upright microscope [20]. 

Recordings were made from striated, multi-nucleated myotubes. Patch pipettes with a resistance of 6–10 MΩ were made from borosilicate glass (BF 150-86-10; Sutter Instrument Company, Novato, CA, USA) with a Sutter P97 pipette puller (Sutter Instrument Company). Current-clamp and voltage-clamp recordings were made utilizing a Multiclamp 700A amplifier (Axon, Union City, CA, USA). The pipette (intracellular) solution contained 140 mM K-gluconate, 2 mM MgCl_2_, 2 mM Na_2_ATP and 10 mM HEPES (pH 7.2). After the formation of a gigaohm seal and membrane puncture, the cell capacitance was compensated. The series resistance was typically <23 MΩ, and it was compensated >60% using the amplifier. Signals were filtered at 3 kHz and sampled at 20 kHz using a Digidata 1322A interface (Axon instrument). Data recording and analysis were performed with pClamp8 software (Axon instrument). Membrane potentials were corrected by subtraction of a 15 V tip potential, which was calculated using Axon’s pClamp8 program. Membrane resistance and capacitance were calculated using 50 ms voltage steps from −85 to −95 mV without any whole-cell or series resistance compensation. The resting membrane potential and depolarization-evoked action potentials were recorded in current-clamp mode. Depolarization-evoked inward and outward currents were examined in voltage-clamp mode. 

### 2.5. Phase Contrast Microscopy and Videography 

Myotube contractions were recorded as pixel differentials with a Hamamatsu digital camera (Model C8484-05G) [18]. The video data were analyzed in Python using the OpenCV library. Briefly, the pixel values of the first frame of the video was subtracted from the pixel values of all subsequent frames, quantifying the degree to which subsequent frames differed from the first. In each experiment, the cells were maintained in N2 media and imaged using an upright Zeiss microscope (Zeiss Hal 100). The videos were recorded using a Hamamatsu digital camera (Model C8484-05G) and analyzed in ImageJ and LabView. The camera was capable of high speed acquisition (50 frames per second) when sampling a subset of pixels such as those corresponding to a contracting myotube. The average pixel intensity from a region of interest (ROI) was plotted in real time to measure contractile amplitude and frequency. A program written in LabVIEW was utilized in controlling the stimulus generator, thereby enabling synchronization with the detector. Muscle contractions in synchronization with stimulations were considered as induced contractions which were different from spontaneous contractions.

### 2.6. Cantilever Fabrication and Force Measurement of Myofiber Contraction 

Cantilever microelectromechanical systems (MEMS) were fabricated from 6-inch silicon-on-insulator wafers, polished with a 5 μm silicon layer and a 500 μm silicon dioxide layer on the front and back respectively using previously published methods [21,22]. The cantilevers were patterned using S1818 photoresist and etched using deep reactive ion etching. These cantilevers were coated with Collagen I (60 μg/mL) before cell plating. Myogenic progenitors were plated onto these cantilevers and myofibers were induced following the procedure as described above for coverslips. 

To test the contraction force of the myofibers grown on cantilevers, the BioMEMs cantilever devices were assembled into engineered acrylic housings at least one day before testing. On testing day, the cultures were transferred to a heated stage, equipped with a light source (above), and photodetector and laser (below). System control and data collection were performed as described by Wilson et al. [21]. Myofibers were tested for their maximum contractile force output (MCF) with a frequency of 0.5 Hz and pulse width of 250 ms, at 5 V. 

### 2.7. Testing the Formation of Functional NMJs 

The ability of iPSC derived myofibers to form functional Neuromuscular junctions (NMJs) was assessed using a dual-chamber NMJ system described by Santhanam et al. [18]. IPSC-derived myogenic progenitors were plated into the muscle chamber of the dual chamber in hSKM growth medium. Myogenic progenitors were grown to confluence by feeding every 2 days, and then switched to HI medium (counted as Day 0) and subjected to myotube induction as described in Section 2.2. Meanwhile, iPSC-derived motoneurons (MNs), differentiated as described in Santhanam et al. [18], were plated in the MN chamber in MN medium. Half of the medium was refreshed every 2 days. Functional NMJ formation was assessed starting from Day 10 of the co-culture. MNs were stimulated at different frequencies (0.33, 0.5, 1, and 2 Hz) while myotube contractions were monitored by videotape recordings of pixel differentials. The video data was analyzed in Python using the OpenCV library. Since these two chambers were chemically and electrically insulated from each other, muscle contractions in synchronization with MN stimulations are considered as induced contractions through NMJ formation. 

## 3. Results

### 3.1. Myofiber Differentiation Observed by Phase Microscopy

**Figure 1:**
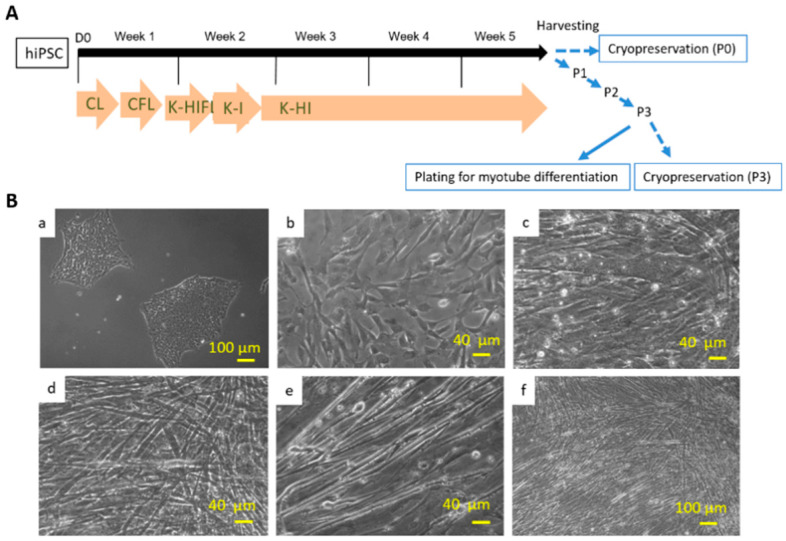
(**A**) Diagram of the myogenic differentiation protocol from hiPSCs. The differentiation protocol from Week 1~5 is basically the same as in Chal’s publication. The culture was then harvested for cryopreservation or 3X series passaging as delineated by the blue arrows, which is the beneficial modification developed in this study. (**B**) Differentiation of myotubes from human iPSCs demonstrated by phase microscopy images. (**a**) iPSCs before myo-lineage differentiation. (**b**) myogenic progenitors before myotube induction. (**c**–**f**) Myotubes at different stages of differentiation and at different magnifications. (**c**) Day 4; (**d**) Day 7; (**e**,**f**) Day 8.

Myogenic progenitors were differentiated from hiPSCs according to the procedures described in Chal et al. [11] and detailed as in the Methods. The entire differentiation process was monitored by phase microscopy (Figure 1). The iPSC culture with scattered colonies (Figure 1A,B) was grown to about 30% confluence before the initiation of differentiation. After the multistep differentiation, which takes 12 days, the culture was maintained in HI medium for another 3 weeks and then either cryopreserved or sub-cultured at lower densities. Differentiated cultures at this stage usually contained a heterogeneous population of cells at various myogenic stages. To obtain a stock of myogenic progenitors of high purity and synchronize myofiber differentiation, an additional passaging procedure was developed. Specifically, the cryopreserved vial or subcultured cells were grown 2 days in HI medium followed by expansion in hSKM growth medium and cryopreserved again after 2–3 passages. Per phase microscopy, the myoblast cultures were mostly a pure population after 2–3 passages in expansion medium, as demonstrated by the uniform morphology (Figure 1B(c,d)). The myofibers were induced from these myogenic progenitors by switching to HI and then N2 medium. Extensive myocyte fusion began to be observed after about 2 days in HI medium, and contracting myotubes appeared after Day 7 in N2 medium (Figure 1B(d–f)). 

### 3.2. Characterization of iPSC-Derived Myogenic Progenitors by Immunocytochemical Analysis

**Figure 2:**
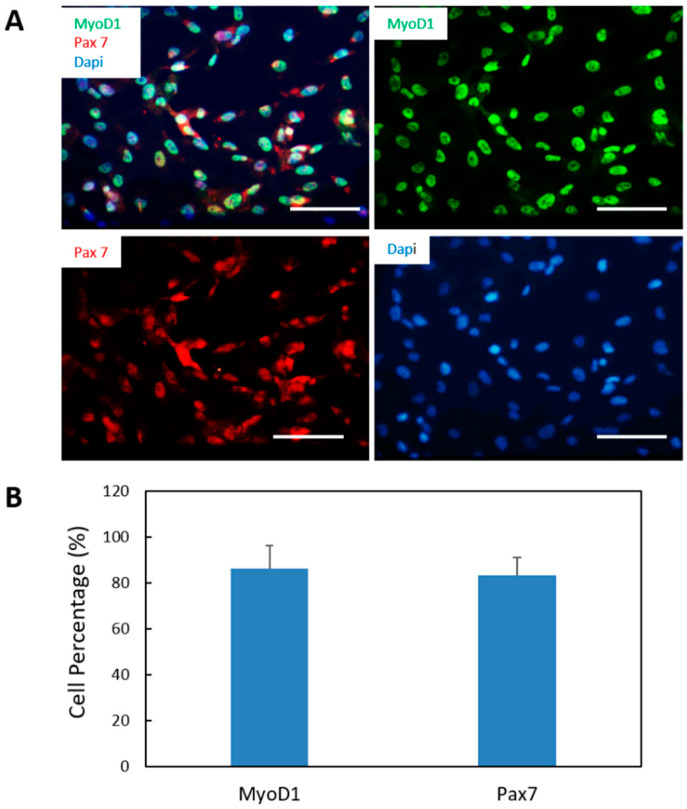
Characterization of myogenic progenitors differentiated from iPSCs. (**A**) Myogenic progenitors differentiated from iPSCs were expanded and immunostained for the myoblast markers MyoD1 (green) and Pax 7 (red). (**B**) Quantification of the percentages of MyoD1-postive cells and Pax7-positive cells out of the total number of cells (visualized by DAPI based on the immunostaining from A. Over 15 images were randomly taken and analyzed for each coverslip. Three coverslips from three different batches of experiments were analyzed. Data presented are mean ± SEM. Scar bars: 100 µm.

Pax 7 marker specifies satellite cells [23] and MyoD1 expression is necessary and sufficient for myogenesis [24]. Co-expression of both markers is associated with activated and proliferative myogenic progenitors [25]. Thus, the expression of Pax7 and MyoD1 was assessed by immunocytochemistry. One vial of myogenic progenitors were plated on coverslips and grown in myoblast expansion medium and evaluated. When the cells reached an appropriate density, the cells were fixed for immunocytochemical analysis. As displayed in Figure 2, cells were positive to both MyoD1 and Pax7, confirming that the cells were myogenic progenitors. Quantification of the immunocytochemistry resultindicated a high purity of myogenic progenitors in culture (MyoD1 86.4 ± 9.9%, PAX7 83.21 ± 7.8%) (Figure 2B). 

### 3.3. Fiber Type Characterization of iPSC-Derived Myotubes 

**Figure 3:**
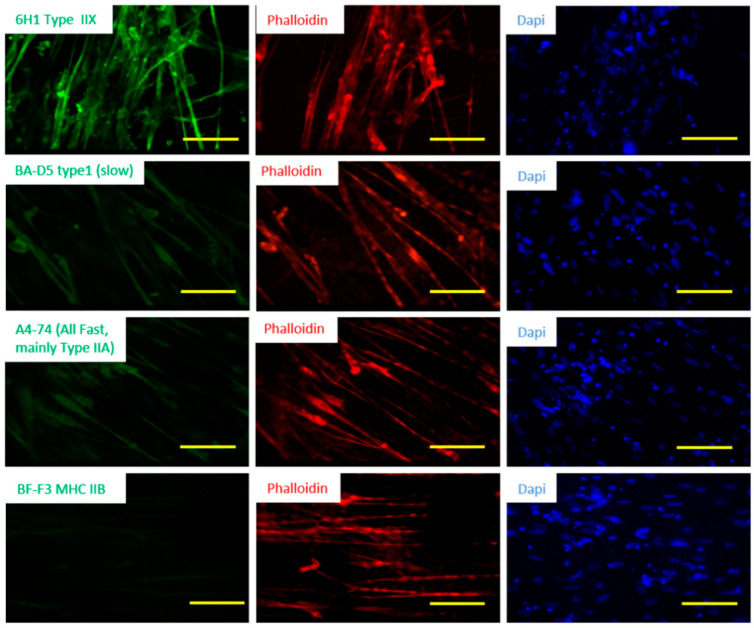
Fiber type characterization of iPSC derived hSKM. The iPSC-hSKM culture after approximately two weeks of differentiation were immunostained with fiber type-specific antibodies which were co-stained with phalloidin to visualize the myotubes. Scale bar: 100 µm.

In mammalian skeletal muscle, multiple fiber types are generally intermingled within a single muscle group, and different muscle groups have varying proportions of fiber types. The proportion of different fiber types in muscle may be a good indicator of muscle physiological properties and pathological status of the muscle [26]. Human muscle usually consists of a combination of the fiber Types I, IIA and IIX, and each is encoded by the genes MHC 7, 2 and 1, respectively. Muscle fiber types are generally defined by the particular myosin heavy chain isoforms that they express, while many other components contribute to a fiber’s physiological characteristics. Type I is a slow oxidative fiber, while Type II are fast contracting fibers in which Type IIA is oxidative and Type IIX is glycolytic [26]. To assess fiber type distribution in the iPSC derived muscle cultures, the myofibers were immunostained with mouse anti-myosin heavy chain (MHC) IIX, MHC IIA or MHC I antibodies. As shown in Figure 3, the iPSC derived myofibers demonstrated the strongest signal for the antibody against MHC IIX (6H1) but weakly for the antibodies against MHC IIA Fast (A4-74) and MHC I slow (BA-D5), and completely negative to MHC Type II B. This observation was the same in the analysis of 3 batches. The expression level of MHC I Slow was not significantly different from that of MHC IIA Fast in general. These results are consistent with in vivo human muscle tissue [27]. For each fiber type analyzed, close to or over half of the Phalloidin-positive myofibers were positive, suggesting that the same population of myofibers stained positive to multiple types of MHCs, or a hybrid composition of MHC subtypes existed in these myofibers. Fibers co-expressing different MHCs have been detected in normal skeletal muscle previously [28].

### 3.4. Characterization of iPSC Myotubes by Immunocytochemistry

**Figure 4:**
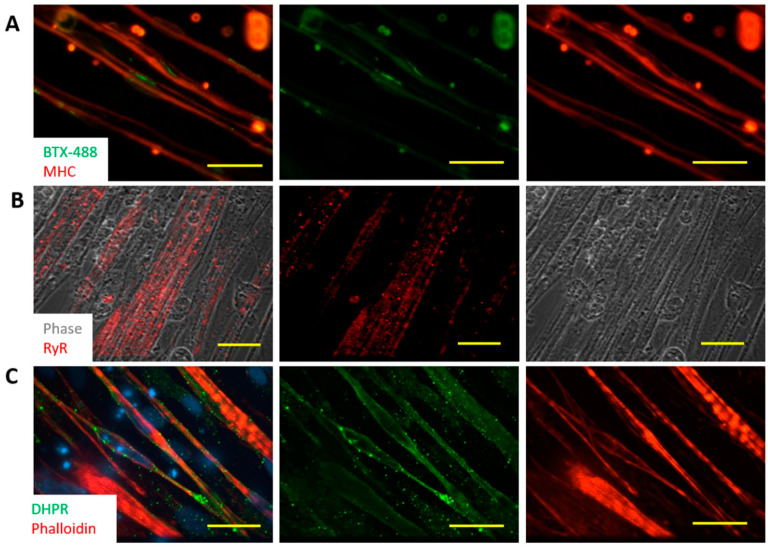
Characterization of myofibers differentiated from human iPSCs. (**A**) Expression of AchRs in a Day 15 SKM culture was demonstrated by staining with BTX-448. Myotubes were visualized with Myosin Heavy Chain (MHC) immunostaining. Scale bar: 100 µm. (**B**,**C**) Immunostaining of these myotubes for Ryanodine Receptor (RyR) (**B**) and Dihydropyridine Receptors (DHPR) (**C**) to examine the formation of excitation-contraction coupling in a Day 15 culture. Scale bars are 25 µm and 50 µm in (**B)** and (**C)**, respectively.

To gain better insight for their functionality, the iPSC derived myotubes were analyzed for the expression of acetylcholine receptors (AChRs) by staining with Bungarotoxin-488 (BTX-488) (Figure 4A). Many BTX-488 clusters were observed on the membrane of the myotubes, indicating widespread expression of AChRs in the myotubes. Expression of AChRs enables the myotubes to respond to ACh, which is the neurotransmitter released by motoneurons. 

The iPSC-derived myotubes were also examined for the development of Ryanodine Receptors (RyR) and Dihydropyridine Receptors (DHPR), which are the essential components of the excitation-contraction apparatus. As in Figure 4B, widespread expression of RyR was observed and their distribution pattern correlated well with the striations seen in the phase images. Similarly, the expression of DHPR in these myotubes (Figure 4C) was ubiquitous and their regular spaced patterns also co-registered with the myotube striations displayed by Phalloidin staining. The expression of RyR and DHPR suggest that these myotubes could convert an action potential into physical contraction, an important step for myotubes to respond to motoneuron innervation. 

### 3.5. Patch Clamp Electrophysiology from iPSC Myofibers

**Figure 5:**
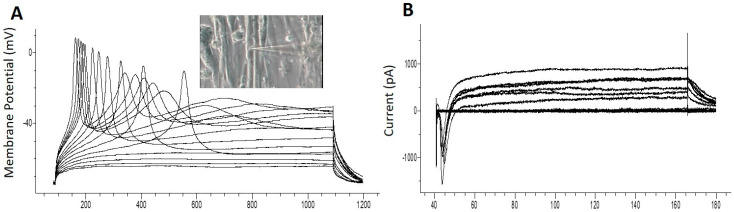
Electrophysiological analysis by patch clamp in Day 10 cultures. After 10 days of differentiation from myogenic progenitors, myotubes were sampled for patch clamp analysis. (**A**) Action potentials induced under current clamp conditions. The inset is an image of the recorded cell. (**B**) Sodium and Potassium currents recorded under voltage conditions.

The electrophysiological competence of the differentiated myotubes was evaluated using patch clamp recordings. The maintenance of a stable resting membrane potential (RP) is indicative of the correct function of multiple ion channels on the myotube membrane, primarily potassium and chloride channels, as well as Na^+^/K^+^ ATPase pumps. It has been reported that myotubes which are not fully differentiated have a less-hyperpolarized RP [29]. The RP reported from cultured human SKM derived from primary satellite cells is −56.3 ± 4.7 mV [30]. RP analysis from these iPSC-myofibers indicated a highly hyperpolarized resting potential at −60.80 ± 1.4 mV, N = 5 (Table 1). This result indicates that the iPSC myofibers are comparable to those sourced from primary satellite cells. Voltage clamp electrophysiology of the myotubes indicated inward and outward currents characteristic of functional sodium and potassium channels (Table 1). Figure 5A provides an example of a single action potential fired by cultured myotubes in current clamp mode, and Figure 5B is an example of current recordings in voltage clamp mode. Taken together, these results indicate that the iPSC derived myofibers were electrophysiologically competent.

### 3.6. Electrical Stimulation Induced Contraction of Human Myotubes in Culture

Although the iPSC derived myotubes spontaneously contracted as those reported for myotubes derived from primary satellite cells [30], and the above data provided strong evidence for the structural basis of ECC in vitro, it is still unknown whether the myotubes were capable of contracting in response to controlled electrical stimulation. To address this question, the contraction of iPSC-derived myofibers was analyze by utilizing two assays.

**Figure 6:**
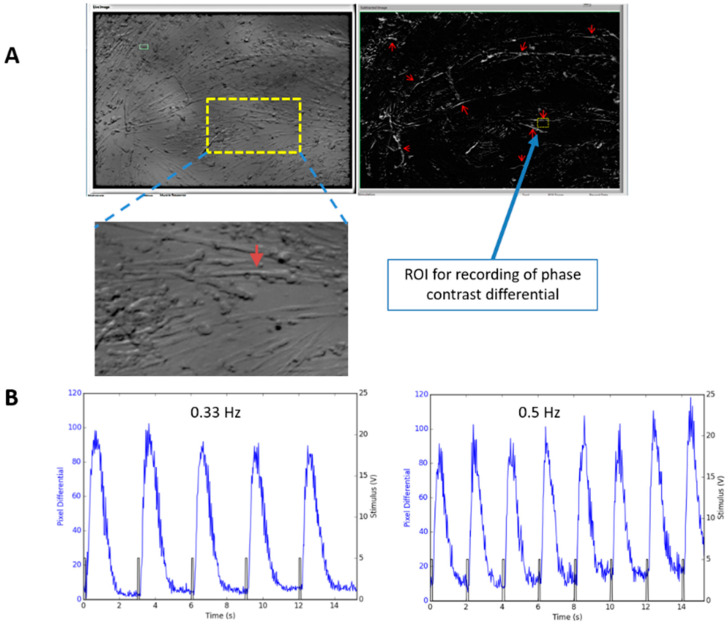
Recording of myotube contraction by measuring pixel differentials under phase contrast microscope. (**A**) Recording of a myotube contraction in a Day 14 culture (D10 in Differentiation) was performed by motion capture of the myotubes using a custom LabVIEW program. Left image in A is a regular phase image. The right is a phase differential image in which all the contracting myotubes “light up” due to the phase contrast change caused by motions. Red arrows point to some of the contracting myotubes where synchronous motion was detected upon stimulation indicating they were contracting. An enlarged image of the recorded myotube was included as an inset. An ROI (Region of Interest) was chosen on one of the contractible myofibers for monitoring myofiber contraction under electrical stimulation. (**B**) Contraction recording from one myofiber under two stimulation frequencies are illustrated as an example.

The first assay was monitoring pixel differentials caused by myofiber contractions visualized utilizing a phase contrast microscope [18]. This method is applicable for analyzing myotube contractions on general 2D surfaces, especially their fidelity to electrical stimulation during increasing frequencies and for various durations. For recording, an iPSC-SKM culture was mounted on the stage of an upright microscope and stimulated to induce contraction. Field stimulation (2 V, duration 40 ms, square wave) with various frequencies (0.33–1 Hz) was applied and the contractions of the myotubes were recorded by measuring the pixel brightness changes caused by the contractions. Figure 6A,B demonstrated that the contraction of these myotubes can be reliably induced by controlled stimulation under the test frequencies. 

### 3.7. Contraction Profiles of iPSC Myotubes Characterized on Cantilevers

**Figure 7:**
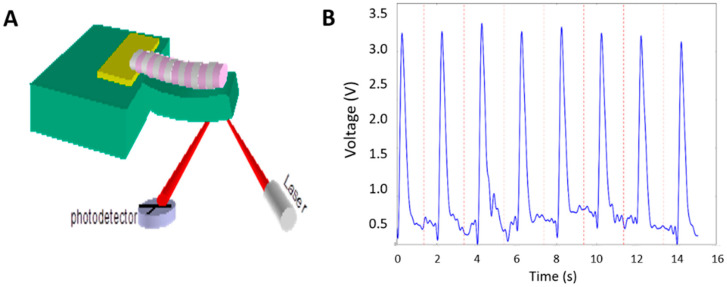
Measurement of iPSC-SKM contraction force on cantilever devices. (**A**) A diagram of the cantilever detection system illustrates that cantilever bending induced by myotube contraction can be detected by laser beam deflection. (**B**) A sample trace of myotube contraction recorded from cantilever bending.

The second assay for analyzing myotube contraction was by utilizing a cantilever device, a Bio-MEMs construct for monitoring myotube contraction (Figure 7A) [22]. This assay requires growing myotubes on cantilevers. It can not only quantify the fidelity of muscle contraction upon each stimulus, but also measure the muscle contraction force. iPSC-myocytes were differentiated to myotubes on a cantilever device using a previously developed platform. The contractions on Days 10–11 of culture were analyzed under controlled field stimulation (0.5 Hz, 250 ms, 5 V) (Figure 7B). Based on recordings and analysis from 20 cantilevers of two batches of experiments, the contraction amplitude of these contractions was 2.94 ± 0.31 V (or 119.8 ± 16.0 nN). These measurements were comparable to those obtained from myotubes generated from primary myoblasts, which were well presented in [31]. These observations provide further evidence for the high level of functional maturation achieved by these iPSC-derived myotubes. 

### 3.8. Innervation of iPSC-SKM by iPSC-MNs from the Same iPSC Line and Formation of Donor-Specific Functional NMJs 

**Figure 8:**
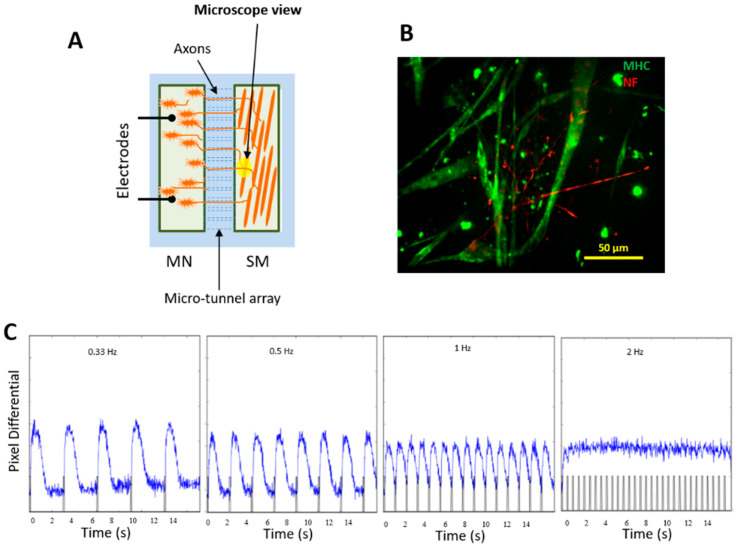
Innervation of iPSC-SKM by iPSC-MNs derived from the same iPSC line. (**A**) A diagram of the NMJ chamber (a courtesy from Santhanam et al. [18]). (**B**) iPSC-derived muscle culture was incorporated into the NMJ chamber system and co-cultured with motoneurons derived from the same iPSC line. Myotubes and MN axons in the muscle chamber were visualized in the muscle chamber through immunostaining utilizing antibodies against MHC (green) and Neurofilament (red) respectively. Analysis was done on Day 12 of the co-culture. The extensive distribution of axonal terminals in the muscle chamber and their physical interactions with myofibers was demonstrated by immunostaining with antibodies against Neurofilament and myosin heavy chain. (**C**) Innervated myofibers contracted in response to motoneuron stimulation under all frequencies tested.

The biological role of skeletal muscle is to respond to MN electrical stimulation and contract. Thus, it was important to investigate the capability of these iPSC-derived skeletal myofibers to form functional NMJs. This capability was assessed in the dual chamber NMJ system [18], in which the muscle chamber and MN chamber are connected through microtunnels which allows the passage of axons. These two chambers are electrically and chemically isolated so that the stimulus in one chamber (whether electrical or chemical) will not affect the cells in the other chamber (Figure 8A). For this experiment, in order to detect the functional NMJ formation, electrical stimuli were applied to the MN chamber while myofiber contractions were monitored in the muscle chamber by phase contrast microscope and a fast video camera. The contractions that synchronized with electrical stimuli were considered as being induced by MN excitation and indicating functional NMJs. IPSC-derived myogenic progenitors and iPSC-derived MNs were co-cultured in this dual chamber, each in its own medium. MNs were differentiated from the same iPSC line as the SKM. MN stock from this iPSC line has a high MN purity and the MNs have been characterized and utilized in multiple published studies, both by immunocytochemistry and for function [17,18,32]. Multiple chambers were tested and functional NMJs were detected in each chamber. As in Figure 8B, immunocytochemistry revealed extensive extension of MN axons into the SKM chamber. Electrical stimulation of the MNs induced myotube contraction locations, indicating sites of functional NMJ formation. Altering the stimulation frequency in a range from 0.33, 0.5, 1 and 2 Hz induced myotube contractions at correspondent frequencies, indicating the high functional fidelity of the NMJs (Figure 8C) [18]. A video is included to demonstrate the recording process and myofiber contractions in correspondence to field electrical stimulations on the MN side at these frequencies (Video S1). The successful innervation of iPSC-SKM by the MNs derived from the same iPSC line indicates the capability of developing patient-specific NMJ models for disease model applications.

## 4. Discussion

This work details the characterization of muscle cells differentiated from human iPSCs by adapting the protocol reported by Chal [11], which mimics the natural differentiation pathway in an attached monolayer of cells in a feeder-free, serum-free system. The structure and function of differentiated myotubes were extensively characterized and the capability of myofiber contraction under electrical stimulation was demonstrated. Most importantly the functional innervation of the iPSC-hSKMs by iPSC-hMNs derived from the same iPSC line was established in a functional NMJ chamber system. Characterization of these iPSC-derived myofibers indicated a level of maturity and the capacity in reproducing multiple functional features similar to primary human skeletal muscle, suggesting a potential for its widespread application for in vitro disease modeling. Utilizing iPSCs enables the transfer of the protocol to iPSCs from different human subjects, which makes it possible to establish patient-specific in vitro models for different diseases.

Phenotypic characterization of the iPSC-SKM indicated these myofibers are equipped with the essential features of primary human skeletal muscle. Morphologically, the myotubes demonstrated the appropriate distribution of the molecular apparatus essential for EC coupling as indicated by positive immunocytochemical identification of RyR and DHPR and BTX staining indicated possible innervation sites (Figure 4). Fiber type analysis revealed a mixture of fast (Type IIx, IIA) and slow (Type I) fiber types and the absence of Type IIB fibers (Figure 3), which is consistent with published analysis from human in vivo samples [27]. Functionally, the iPSC-SKMs demonstrated competent functional electrophysiology, capability of contracting upon electrical stimulation, and with a contracting force comparable to myofibers derived from primary myoblasts. Most importantly, these myofibers could be innervated by human MNs which were derived from the same iPSC line. All these characteristics indicated that these iPSC-SKM have comparable structural and functional capacity to those derived from primary tissues. 

This functional in vitro human skeletal muscle system could provide a powerful tool for research in multiple fields. It offers a valuable platform from which to investigate human muscle development and physiology, muscle-related diseases and pathology while being potentially compatible with future high content drug development and toxicology investigations. The defined nature of the culture protocol facilitates the application of this system in mechanism dissection and enhances its reproducibility. The contractile measurements of these cultured human myotubes demonstrated here also significantly expands the scope of their application in fields of study focused on muscle kinetics, metabolism, and sports-related muscle physiology. The functional myotubes can also be co-cultured with motoneurons in order to study NMJ-related human physiology and etiology [18,33]. This defined, human-based, iPSC-derived in vitro muscle system, as well as the patient-specific NMJ system, have great and wide-reaching potential applications in the fields of tissue engineering, patient-specific drug testing, high content drug screening and the investigation of musculoskeletal system physiology and disease.

## Figures and Tables

**Table 1:** Quantification of the electrophysiological parameters (N = 5 cells). RP: Resting membrane potential; INa^+^: Sodium current; IK^+^: Potassium current; AP: Action Potential; Rm: Membrane Resistance; Cm: Membrane Capacitance.

	RP(mV)	INa^+^ (pA)	IK^+^ (pA)	AP (mV)	Rm (MΩ)	Cm (pF)
Average	−60.80	1128.85	633.20	107.10	214.25	196.60
SEM	1.40	211.91	100.81	21.85	18.31	9.04

## Supplementary Materials

The following are available online at https://www.mdpi.com/2306-5354/7/4/133/s1, Video S1: Recording myofiber contractions induced by motoneuron stimulation.
